# P-1201. Impact of Demographic Factors and Social Determinants of Health on RSV Immunoprophylaxis in Infants

**DOI:** 10.1093/ofid/ofae631.1383

**Published:** 2025-01-29

**Authors:** Patrick J Reich, Tyler Walsh, Derek Harford, Angela Niesen, Caren Liviskie, Brandy Zeller, Ana Maria Arbelaez, Beverly Brozanski, Melissa Riley, Barbara Warner, Jason G Newland

**Affiliations:** Washington University School of Medicine, Saint Louis, Missouri; Washington University School of Medicine in Saint Louis, St. Louis, Missouri; St. Louis Children's Hospital, Saint Louis, Missouri; St. Louis Children's Hospital, Saint Louis, Missouri; St. Louis Children's Hospital, Saint Louis, Missouri; St. Louis Children's Hospital, Saint Louis, Missouri; Washington University, St Louis, Missouri; Washington University School of Medicine, Saint Louis, Missouri; Washington University School of Medicine, Saint Louis, Missouri; Washington University School of Medicine, Saint Louis, Missouri; Washington University in St. Louis School of Medicine, St. Louis, Missouri

## Abstract

**Background:**

In 2023, two highly effective forms of respiratory syncytial virus (RSV) immunoprophylaxis to prevent severe RSV disease in young children were approved: Abrysvo (vaccine in pregnancy) and nirsevimab (infant monoclonal antibody). However, challenges with supply and insurance reimbursement limited widespread availability during the 2023-2024 RSV season.

**Table.**
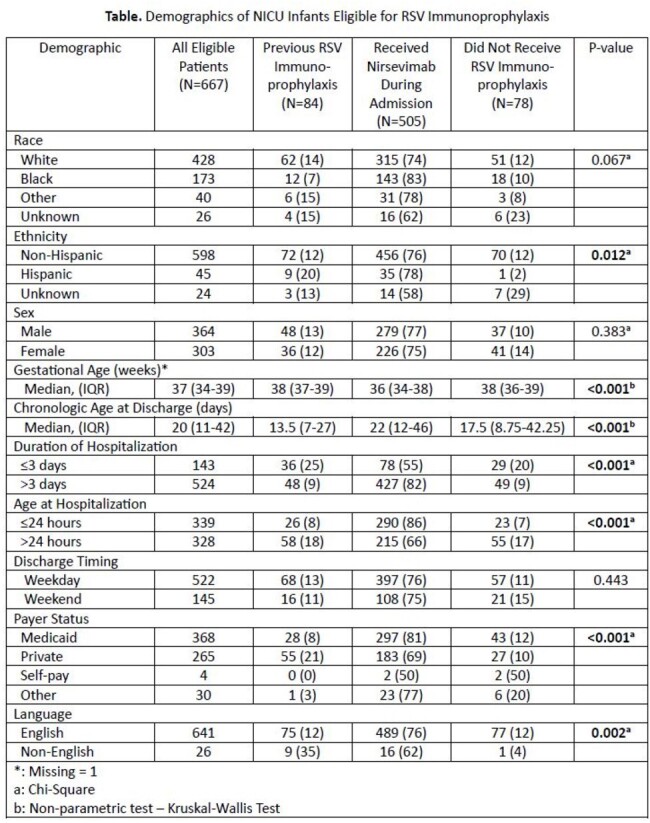
Demographics of NICU Infants Eligible for Immunoprophylaxis

**Methods:**

From October 9, 2023 through March 31, 2024, all St. Louis Children’s Hospital Neonatal Intensive Care Unit (NICU) discharges were offered nirsevimab with no out-of-pocket cost, per CDC recommendations to prioritize infants at highest risk. Data abstraction followed by statistical analyses were performed to identify differences in demographic factors between the following groups:

Group 1: Infants who received RSV monoprophylaxis (infant nirsevimab or maternal Abrysvo) prior to hospitalization

Group 2: Infants who received RSV immunoprophylaxis (nirsevimab) during hospitalization

Group 3: Infants who did not receive RSV immunopropylaxis

Area Deprivation Index (ADI) maps were generated using ArcGIS Pro to identify the proportion of infants in the above groups from zip codes with higher or lower ADI.

Area Deprivation Index for Patients who Received Previous RSV Immunoprophylaxis

**Figure:**
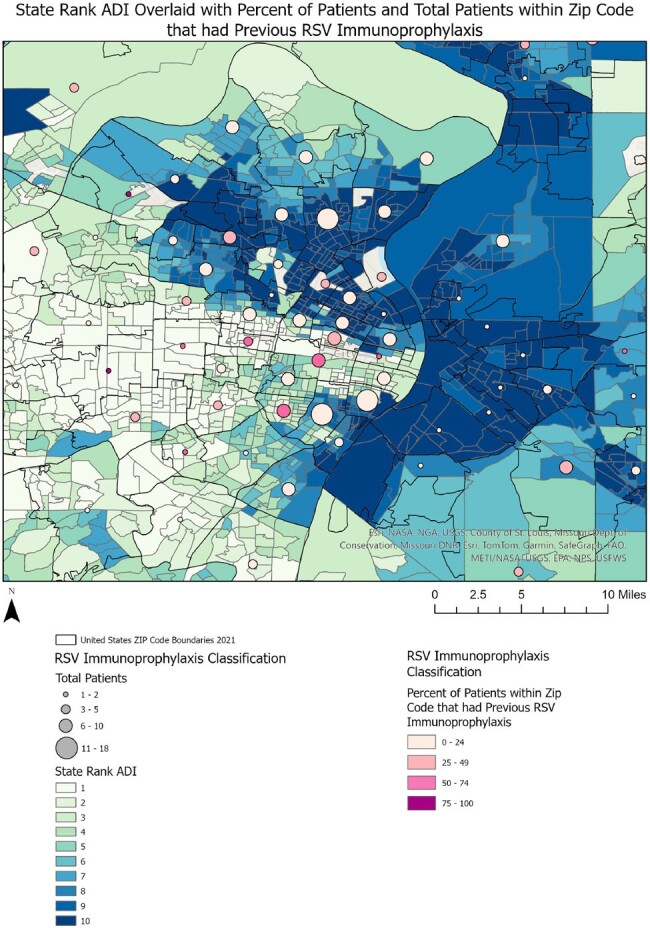

The state rank area deprivation index (ADI) overlaid with the total number and percentage of patients within that zip code who received RSV immunoprophylaxis prior to their NICU hospitalization.

**Results:**

Of 667 eligible infants, 88% received RSV immunoprophylaxis prior to/during hospitalization. Differences in gestational age at birth, chronologic age at hospitalization, age at discharge, duration of hospitalization, and insurance type were observed between groups (Table). Infants who received immunoprophylaxis prior to their hospitalization were primarily clustered in areas with lower ADI (Figure 1). Infants who received immunoprophylaxis during their hospitalization were from areas with variable ADI (Figure 2). Infants who did not receive immunoprophylaxis were primarily clustered in areas with higher ADI, indicating higher social vulnerability (Figure 3).

Area Deprivation Index for Patients who Received RSV Immunoprophylaxis During NICU Hospitalization

**Figure:**
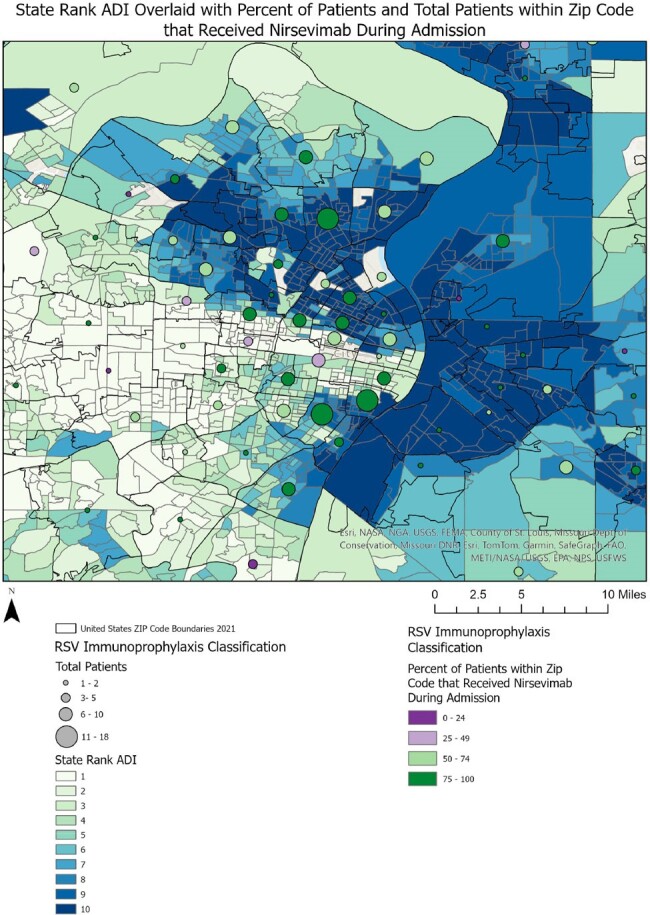

The state rank area deprivation index (ADI) overlaid with the total number and percentage of patients within that zip code who received RSV immunoprophylaxis during to their NICU hospitalization.

**Conclusion:**

Differences in demographics and ADI were identified in infants who received RSV immunoprophylaxis prior to or during their NICU hospitalization compared to those who did not. Future strategies to improve access and availability of RSV immunoprophylaxis that consider social determinants of health are critical to increase coverage and prevent severe RSV infections in infants.

Area Deprivation Index for Patients who Did Not Receive RSV Immunoprophylaxis

**Figure:**
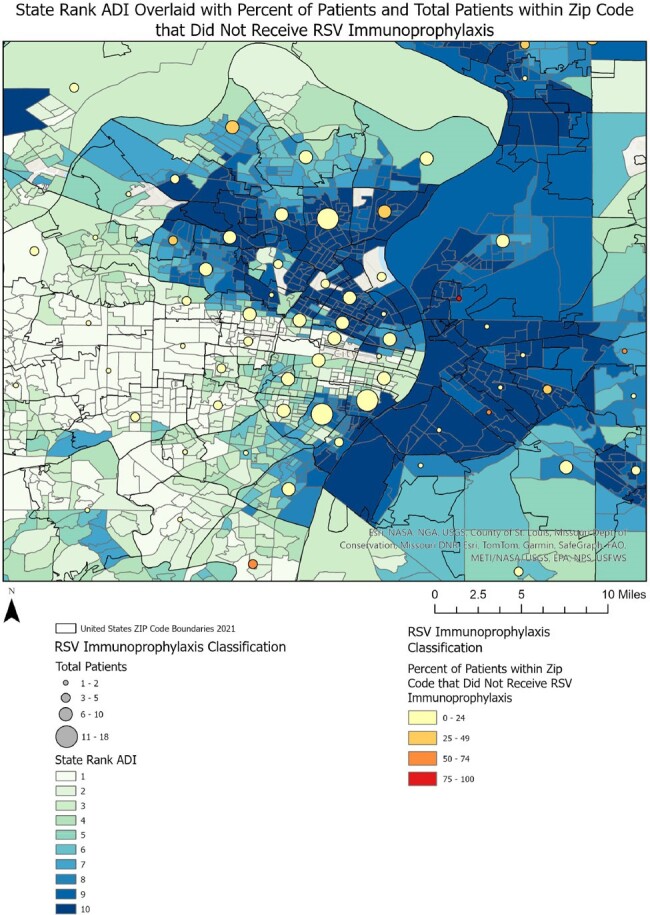

The state rank area deprivation index (ADI) overlaid with the total number and percentage of patients within that zip code who did not receive RSV immunoprophylaxis.

**Disclosures:**

**Jason G. Newland, MD, MEd**, Moderna: Grant/Research Support|Pfizer: Grant/Research Support

